# Ups and downs in physician - cancer patient relationship

**Published:** 2015

**Authors:** M State, I Briceag, B Popescu, R Scăunașu, P Armean

**Affiliations:** * ”Carol Davila” University of Medicine and Pharmacy, Bucharest, Romania; ** E.N.T. Department, “Coltea” Clinical Hospital, Bucharest, Romania; *** General Surgery Department, “Coltea” Clinical Hospital, Bucharest, Romania

**Keywords:** autonomy, legal implications, dialogue models, ethics

## Abstract

**Introduction.**Over the past years, there has been an increasing interest in involving the cancer patients in the decision making regarding the therapy management due to several factors. The most important aspect to be taken into consideration is the principle of patient autonomy. More and more patients have become interested in making informed decisions regarding their therapy options and physicians need to be able to provide data on the aspect. Some patient-physician models have been proposed and used for 40 years now. Still, the debate is very important for most of the physicians due to the shifts in the approach.

**Materials and methods.** To really express the concerns that the authors address, the case of a head and neck cancer patient and the possible dialogues with the physician were presented. Each type of communication model with the patient is very important because nowadays, intrications between the four are likely to occur. There are legal aspects that need to be taken into consideration such as the informed consent, the ethical and moral aspects.

**Conclusions** The possibility of individualized oncological therapy for the cancer patient leads to different decisions for both the patient and the physician. The decision making process involves the patient at different levels. Legal implications tend to affect the healthiness of the dialogue between the physician and the cancer patient and might be a key-point in the further development of the ethical aspects.

## Introduction

Over the past years, there has been an increasing interest in involving the cancer patients in the decision making regarding the therapy management due to several factors. The most important aspect to be taken into consideration is the principle of patient autonomy. More and more patients have become interested in making informed decisions regarding their therapy options and physicians need to be able to provide data on the aspect. Some patient-physician models have been proposed and used for 40 years now. Still, the debate is very important for most of the physicians due to the shifts in the approach. In modern medicine, a patient is entitled to self-decision, an attribute that is called autonomy. This is a concept that has been studied over the past decades in order to establish the ethical principles regarding medical care. In our case, cancer patients are those involved in the therapy management and due to the implications of the life altering disease, the patients need to express their will after being presented the facts. It is a common medical practice to give the patients an overview of the disease, cancer, and all the data necessary for him/ her to make a decision. Evidence based medicine is the way to evaluate benefits and risks in all the medical cases. This is why a healthy physician-cancer patient relationship needs to be a two way street, both parties being involved in the decision making process. Commonly, there are four types of relationship considered between a health care practitioner and a patient. This has been stated in a comprehensive work by Emanuel & Emanuel, in 1992, based on research in healthcare services dynamics in the 1980s and 1990s [**1**]. They discussed the need for redefining the relationship between a physician and a patient, based on the active involvement of the patient in the entire medical process and playing an active role in the treatment decisions. One way to address a patient from a physician’s point of view is the paternalistic model. This model has also been named priestly or parental [**2**,**3**]. This has been the main type of relationship between a physician and a cancer patient in Romania in the past 5-6 decades. By using this type of dialogue, physicians play an active role in deciding the treatment for each individual, acting like a guardian of the patient [**4**]. Usually, patients have a tendency to address a particular healthcare practitioner based on the skills, fame, notoriety and medical aptitudes. This being said, it is easy to understand why most patients do whatever their physician tells them to do. However, the physician has to present all the data regarding the diagnostic procedures and therapy management to the patient in order to have an informed consent from the patient. These data will encourage the patient to follow the physician’s idea of the treatment. There are certain situations in which the physician informs the patient on the medical solutions with authority. This is nowadays considered an ineffective way to address a patient and more, it is outside the law. Some ethical principles need to be taken into consideration when speaking about the treatment with a cancer patient, one of which being self-determination. A physician also needs to take into consideration the patient’s right for justice. These ethical principles have been thoroughly discussed after The Second World War and resulted in different codes and laws, such as The Nuremberg Code 1947, The Declaration of Helsinki 1964 and The Belmont Report 1979 [**5**]. The informative type is at the opposite site of the physician-patient relationship because of the fact that the physician tells the patient all the treatment options, benefits, risks, alternatives, prognosis, follow-up and another relevant medical information, but the patient is the one who decides what he/ she thinks is best for him/ her. This type of relationship is based on some medical knowledge from the patient in order for the patient to fully understand the treatment options. This is a tricky part of the dialogue with the patient because, in Romania, most of the patients do not have a medical education and most of the data regarding different diseases, in our case malignant disease, are gathered from non-professionals. One of the modern aspects of this problem is internet medicine. More and more patients have access to data on the internet from untrusted and mainly non-professional sites. This is why physicians need to allocate increasingly more time to explain the errors in these data to the patients. Form our practice we find that a suited way to talk to the cancer patients is a combination between the two types of relationships presented, paternal and informative. After expressing the medical data the physician is entitled and has to give a solution to the patient’s problem but he/she need to take into consideration the patient’s will. This self-determination of the patient has to be informed and the patient has to be guided into making the right decision. The two other models of relationship, the interpretive model and the deliberative model, are less used in medical practice in Romania. Still, these options have to be explored by the physicians due to the different values for each patient. The interpretive model sets the physician into helping the patient explore personal values and choose a treatment that best fits these values [**1**]. The deliberative model is similar to the interpretive model, the difference being made by the exploration of the medical related values. The patient is the one who decides upon the treatment. There is a tendency of shifting the relationship towards an informative type because of the type of medicine that is nowadays practiced, meaning evidence-based medicine. The internet plays an important role in this type of shifting as discussed before. Still, information is available for the patients in different forms. Correct medical information is the underlying condition for a patient to express a medical opinion even if this opinion affects the individual. The physician has to take into consideration the will of the patient for a third party decision. According to the medical law, this is the case when a patient asks the physician to discuss therapy options and makes decisions with a designated person. This person may be a relative, a friend or any other individual the patient indicates to the physician. Still, the physician is obligated not to withhold important medical data from the patient or a third party pointed by the patient even if the physician considers that disclosing certain data is medically contraindicated. This is known as “therapeutic privilege” and the absence of this is ethically unacceptable [**1**]. This consists of building a conflict of interests between the patients’ welfare and the obligations of the physician. The way of eliminating this type of conflict is by the informed consent. The most valid form of the informed consent is in the form of a dialogue between the physician and the patient in a private room with media devices capable of recording the conversation. At the end of the discussion, both the physician and the patient need to sign a consent form in which the physician needs to state that the patient understood all the medical data presented and the patient needs to state that he/ she understood the medical facts. This is sealed with signatures from both the physician and the patient. Not all information need to be communicated to the patient immediately or all at once; physicians should assess the amount of information a patient is capable of receiving at a given time, delaying the remainder to a later, more suitable time, and should tailor the disclosure to meet the patients’ needs and expectations in light of their preferences [**6**]. Physicians may consider delaying disclosure only if early communication is clearly contraindicated. Physicians should continue to monitor the patient carefully and offer complete disclosure when the patient is able to decide whether or not to receive this information. This should be done according to a definite plan, so that disclosure is not permanently delayed [**6**].

## Materials and methods

To really express the concerns that the authors address, the case of a head and neck cancer patient and the possible dialogues with the physician were presented. Each type of communication model with the patient is very important because nowadays intrications between the four are likely to occur. There are legal aspects that need to be taken into consideration, such as the informed consent, ethical and moral aspects.

A 55-year-old male, from urban environment, heavy smoker, presented to the E.N.T. emergency room with dysphonia, dysphagia, odynophagia and a tumor mass in the right lateral region of the neck, with an insidious onset, 6 months prior and with a progressive evolution. Clinical examination raised the suspicion of larynx and pharynx malignant neoplasia with regional lymph node metastasis in the right lateral region of the neck. Imaging studies sustained the diagnosis with no other distant site metastasis. Biopsy from the larynx set the diagnosis, squamous cell carcinoma of the larynx. The patient was presented with the medical data regarding the diagnosis.

The dialogue with the patient was conducted by the physician in the examination room and, with the will of the patient; the wife was invited to participate. The treatment options were presented to the patient, which is total laryngectomy with functional bilateral neck dissection, permanent tracheostomy as a first line treatment. The benefits and the risks, incidents and accidents were presented to the patient as well as the alternative treatments, radiation therapy and chemotherapy. In addition, statistical data and guidelines for this particular type of cancer were presented. The patient’s reaction was of desperation so that the physician had to calm the patient down and walk him to the diagnosis and treatment options. Because of the way of life of the patient and his denial, the physician asked the patient to think to everything that had been discussed, write down whatever additional questions he might have, talk to the family and communicate his decision in a few days. After 4 days, the patient came to the physician and consented to the surgical therapy.

## Discussions

Our experience with cancer patients and their needs made us address the dialogue from a combination of the paternalistic and informative type. The right of self-determination is very important from a personal, ethic, moral and legal point of view.

The paternalist model relies on the knowledge of the physician and his will to act in the best interest of the patient. The physician will act according to what he/ she thinks is best for the patient, leaving the patient with no other treatment options. When talking about cancer patients, it is very important to help the patient decide what is best for him/ her after correctly informing the patient about all the aspects of the disease. Family and friends play a crucial role for Romanian patients and they need to be involved in the decision-making and also in supporting the patient.

Physicians mainly use goal-oriented rationality and patients mainly value oriented rationality, but in the case of non-curative treatment refusal, physicians give more attention to value oriented rationality. A consensus between the value-oriented approaches of the patient and the physician may then emerge, leading to the patient’s decision of being understood and accepted by the physician [**7**].

## Conclusions

In the past years, talks about the physician-patient relationship have been focusing on two directions, paternalism and self-determination. This was a turning point in the relationships between the two parties involved in the therapy decision making, very much because many have blamed the paternalist relationship used by the physicians empowering themselves over the patients, thus taking control over the decisions. Legal matters and moral issues have taken the perspective to an informative point of view, also dominant in medical ethics. The empowerment of the patient reduces the role of the physician to a counselor of medical matters and eventually to a practitioner if the situation requires it. This is a defective perspective because the physician has to express the understanding, knowledge of the medical condition, well-determined action and capability, psychology and above all, integration for all. The speech of the physician has to persuade the patient to act in his/ her best interest concerning the patient’s values and morals.

In the end, we might find that certain models, one or more of the four, might not be effective in particular cases. This is the time for a caring attitude and the people’ skills, which every physician possesses.

**Acknowledgement**

This paper is supported by the Sectoral Operational Programme Human Resources Development (SOP HRD), financed from the European Social Fund and by the Romanian Government under the contract number POSDRU/159/1.5/s/137390/.
